# Effect of space diffuser on flow characteristics of a centrifugal pump by computational fluid dynamic analysis

**DOI:** 10.1371/journal.pone.0228051

**Published:** 2020-02-03

**Authors:** Ying-Yuan Liu, Gang Yang, Ying Xu, Fan Peng, Le-Qin Wang

**Affiliations:** 1 The College of Information, Mechanical and Electrical Engineering, Shanghai Normal University, Shanghai, China; 2 Institute of Process Equipment, Zhejiang University, Hangzhou, China; Coastal Carolina University, UNITED STATES

## Abstract

Achieving an optimal configuration of the diffuser is indispensable for high pump performances. In this work, a numerical study on diffuser configuration is conducted for a high pump performance using a computational fluid dynamics code, and the effects of the wrap angle and the relative position of the diffuser vane to the impeller on pump performances are included. The results indicate that the modified diffuser with a suitable wrap angle may improve the pump hydraulic efficiency and the head by approximately 4% and 8%, respectively, while a suitable position of the diffuser vane can enhance the pump head by more than 4%. Meanwhile, the pressure recovery coefficient and the local Euler head of the diffuser are adopted to evaluate the diffuser performance. For a high pump performance, the local Euler head of the diffuser has a peak value at the leading edge with the change rate of zero along the meridian streamline, meaning that no blade loading at the leading edge of the diffuser guarantees a better match between the impeller and the diffuser.

## 1. Introduction

Due to the advantage of a large axial length and a small radial length, centrifugal pumps with a space diffuser are widely employed in nuclear power plants, solar concentrating power stations, and many other industrial applications. With the increasing demand in industrial areas, the space diffuser type pumps are facing higher design requirements, i.e. high hydraulic efficiencies and high pump heads [1]. However, diffuser vanes are very distorted and difficult to design, lacking mature theories and experiences. Meanwhile, it is a well-known fact that the diffuser of a centrifugal pump plays a vital role in the energy transformation leading to better pump head and efficiency [2]. Thus, it is significant and necessary to explore an optimal structural configuration of a space diffuser for an improved performance.

With the development of the computer technology, computational fluid dynamics (CFD) is proved to be powerful for the optimal design of pumps and there are several optimal design techniques for pump performances based on CFD. Most of these works focus on the core component of the pump, e.g. the impeller [3–4]. Influences of various structural parameters of the impeller on flow characteristics of the pump are studied, including the blade angle, the wrap angle, the impeller diameter, the inlet/outlet width, the blade fillet, and so on. The results demonstrate that these structural factors present a certain effect on flow characteristics of the pump, where wrap angles of impeller blades have a substantial effect on the flow performance of the pump [5]. Beside the impeller of the pump, there are some other structural features, e.g. the diffuser, which may also enhance the flow behavior of the pump. The diffuser is usually installed downstream from the impeller, with the similar geometrical configuration to the impeller. It serves the following purposes more than a volute: (1) collecting the liquid from the impeller and sending it to the lower impeller inlet or outlet pipeline; (2) eliminating the velocity of the rotation component and transforming the velocity energy to the pressure energy, which is controlled by the blade angle of the diffuser. Regarding the optimal design of the diffuser, two kinds of attempts are employed to improve the performance of fluid machinery: one attempt is to study the effects of critical geometrical parameters on the pump performance using commercial CFD codes. For example, Boncinelli, et.al [6] analyzed the effects of the meridional channel geometries and the blade turning angles of a bowl-type diffuser for a low specific-speed pump to achieve an optimal configuration. Bai, et.al [7] investigated the effect of diffuser vane numbers on the performance of a centrifugal pump with a diffuser and concluded that a lower number of diffuser vanes is beneficial to obtain a good pump stability. It should be stated that some critical geometrical parameters of the diffuser are not mentioned, such as the wrap angles and the vane positions of the diffuser. The other attempt is to design and optimize a diffuser by the inverse design method. Different from the parametric studies given above, the effects of the geometrical parameters of the diffuser do not need to be analyzed by CFD and the blade loading can be adjusted directly for a high performance of diffuser during the inverse design process [8–9]. Results show that an improved structural configuration of the diffuser can be gained by adjusting the blade loading and the corner separation is suppressed using fore loading at the hub of the diffuser. For the inverse design method, the accurate blade loading distribution of the diffuser should be clearly established for a higher performance.

Based on this, the effects of critical geometrical parameters (e.g. the wrap angle and the array position of the diffuser vane) of the diffuser on pump performance are investigated by a CFD code for an intuitive understanding of improving the performance of the centrifugal-flow pump. Meanwhile, the pressure recovery coefficients and the local Euler heads (related to blade loadings) of various diffuser designs are adopted to evaluate the performance of the pump, providing some guidance for the inverse design of the diffuser. The structure of this work is arranged as follows: Section 2 introduces the geometric model and the structural parameters of the centrifugal pump. The numerical model and the analysis strategy for the pump model are described in Section 3, verified by experimental results. Main factors related to the diffuser are discussed in Section 4, including the wrap angle and the array position of the diffuser vane. Meanwhile, the pressure recovery coefficients, the hydraulic losses and the local Euler heads of the diffuser vanes are analyzed to explain the influences of blade wrap angle and position, respectively. Conclusions drawn through CFD investigations are given in Section 5.

## 2. Geometric model and parameters

### 2.1 Geometric model

**Fig 1:**
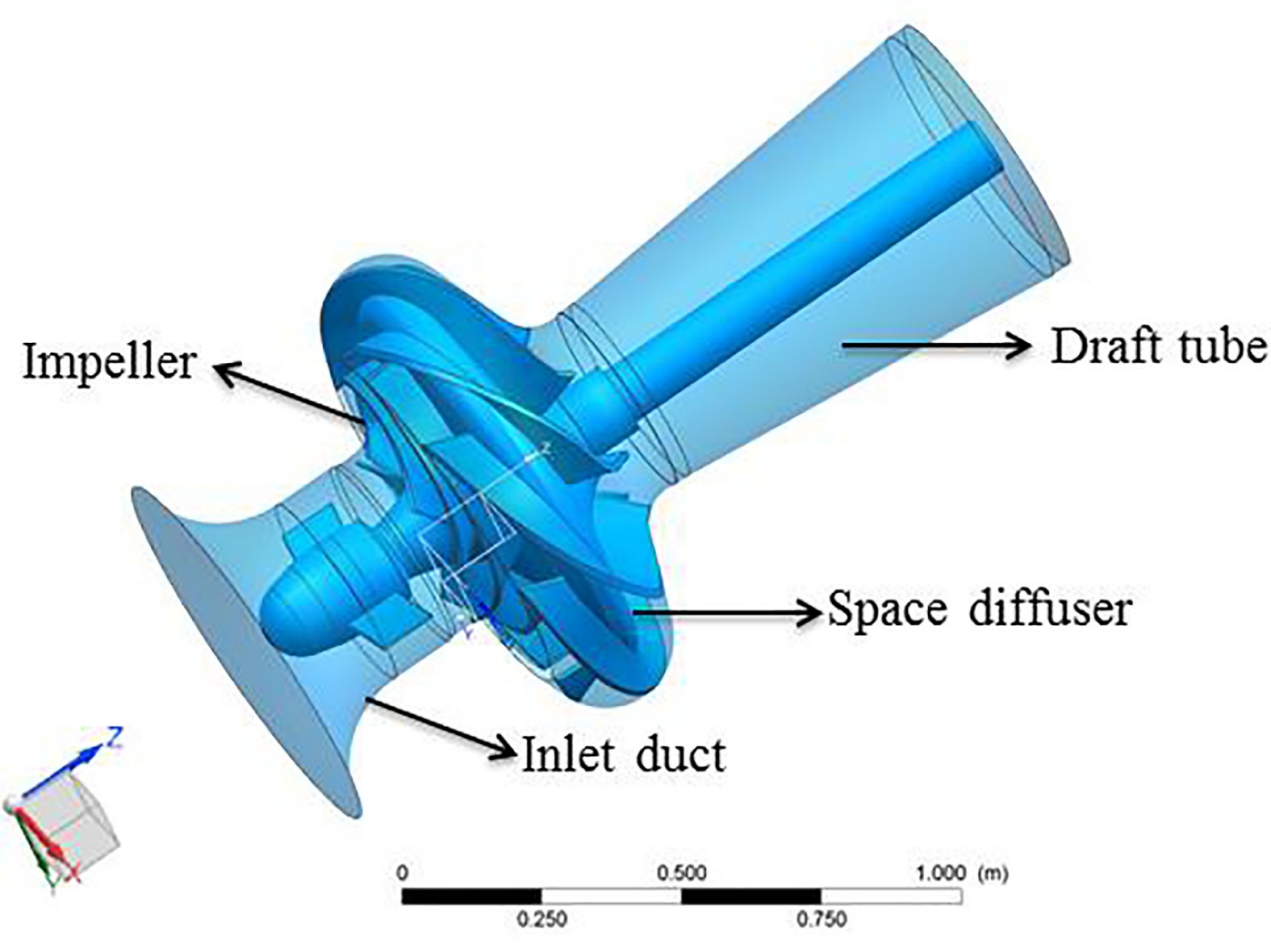
Scheme of the centrifugal pump with a diffuser.

Fig 1 shows the geometric model of the vertical centrifugal pump of a dimensionless design specific speed of 158 ($3.65n\sqrt{Q}/H^{3/4}$), where *n* is the rotational speed of the impeller with unit (rpm), *Q* is the volume flow rate at the best efficiency point (BEP) with unit (m^3^/s), and *H* is the head at BEP with unit (m). The BEP and the main geometrical parameters of the pump are reported in Table 1. To obtain a more accurate solution, the 3D full passage of the pump was modeled, including the inlet duct, the impeller, the space diffuser and the draft tube. The space diffuser was installed downstream from the impeller.

**Table 1:** Best efficiency point (BEP) and main geometrical parameters of the model.

Parameters	Values
Flow rates (m^3^/h)	2858.59
Rotational speed (r/min)	990
Total head (m)	55.54
Outlet Diameter of impeller *D*_2_ (mm)	720
Inlet Diameter of impeller *D*_1_ (mm)	392
Outlet width (mm)	75
Number of blades	6
Inlet Diameter *D*_3_ of the diffuser (mm)	722
Number of vanes	7

### 2.2 Structural parameters

**Fig 2:**
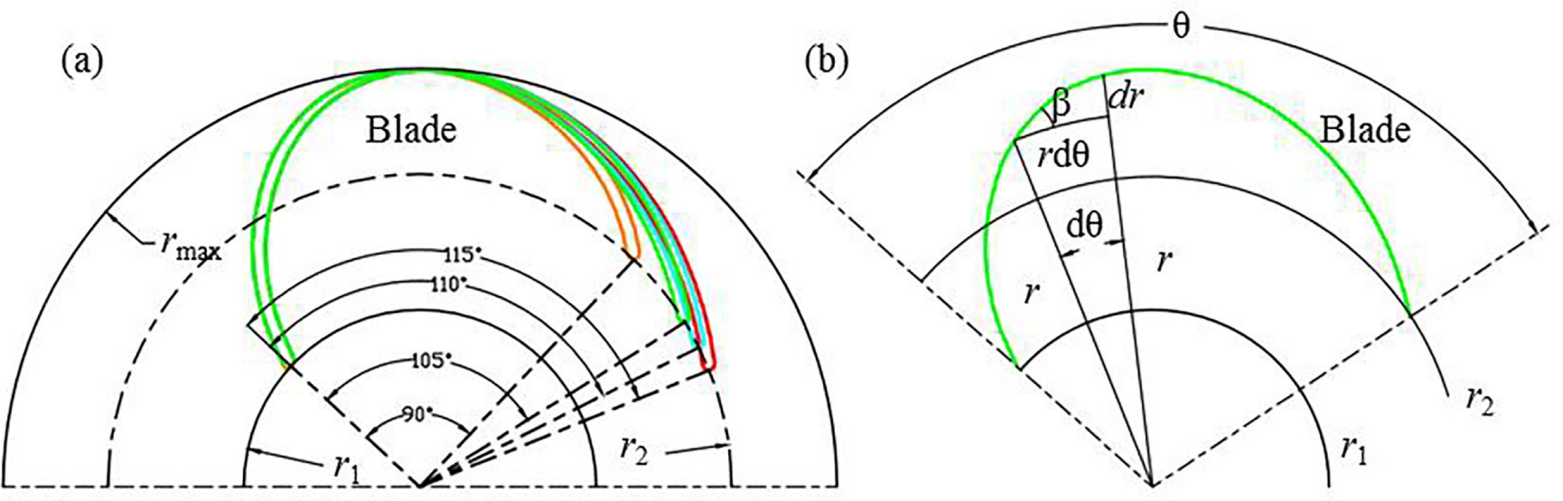
Blade wrap angle: (a) definition and (b) relationship between *θ* and blade angle *β*.

Fig 2 presents the definition of the wrap angle of the diffuser. In case of an infinitesimal increase of circumferential angle d*θ*, the tangent of the flow angle *β* at radial coordinate *r* is shown in Eq (1) and then the wrap angle of the diffuser can be gained by Eq (2) [1]. It can be observed in Eq (2) that the wrap angle is closely related to the blade angle and the radius.

In this paper, the warp angle and the location of the diffuser vane are adjusted through the ANSYS Design Modeler. For the wrap angle of the diffuser vane, the blade angular coordinate of the leading edge (LE) of the diffuser is adjusted to analyze the effect of the wrap angle. Correspondingly, the inlet blade angle is also changed but is not displayed here. For convenience, the wrap angle value on the hub layer is adopted to describe the difference of the wrap angle, with the values of 90°, 100°, 105°, 110° and 115°.

$$\tan(\beta (r))=\frac{dr}{rd\theta }$$ (1)

$$\theta =\int_{r_{1}}^{r_{2}}\frac{1}{r\tan(\beta (r))}dr$$ (2)

**Fig 3:**
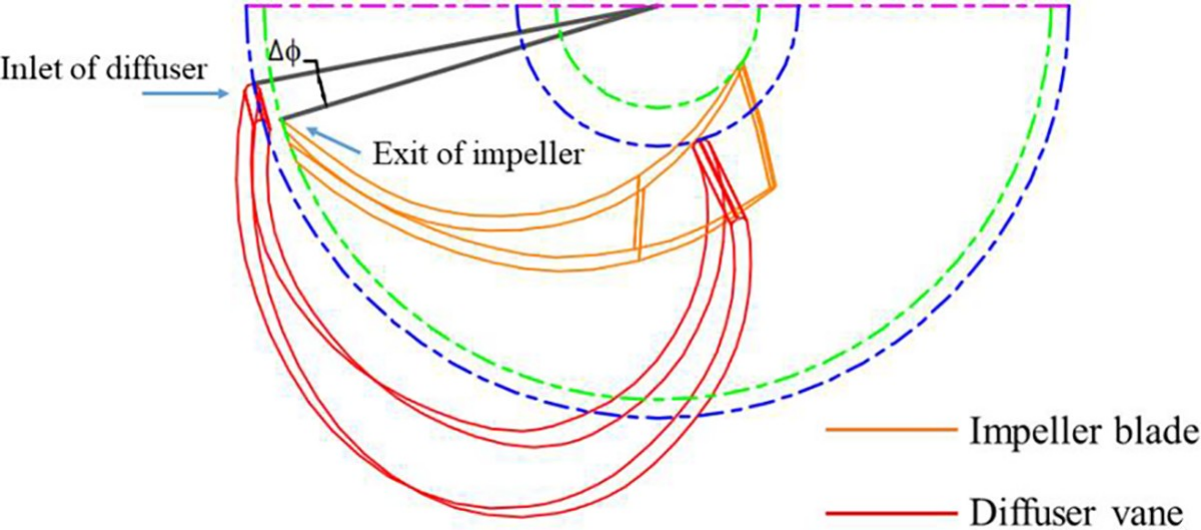
Relative position between the blade of the impeller and the vane of the diffuser.

**Fig 4:**
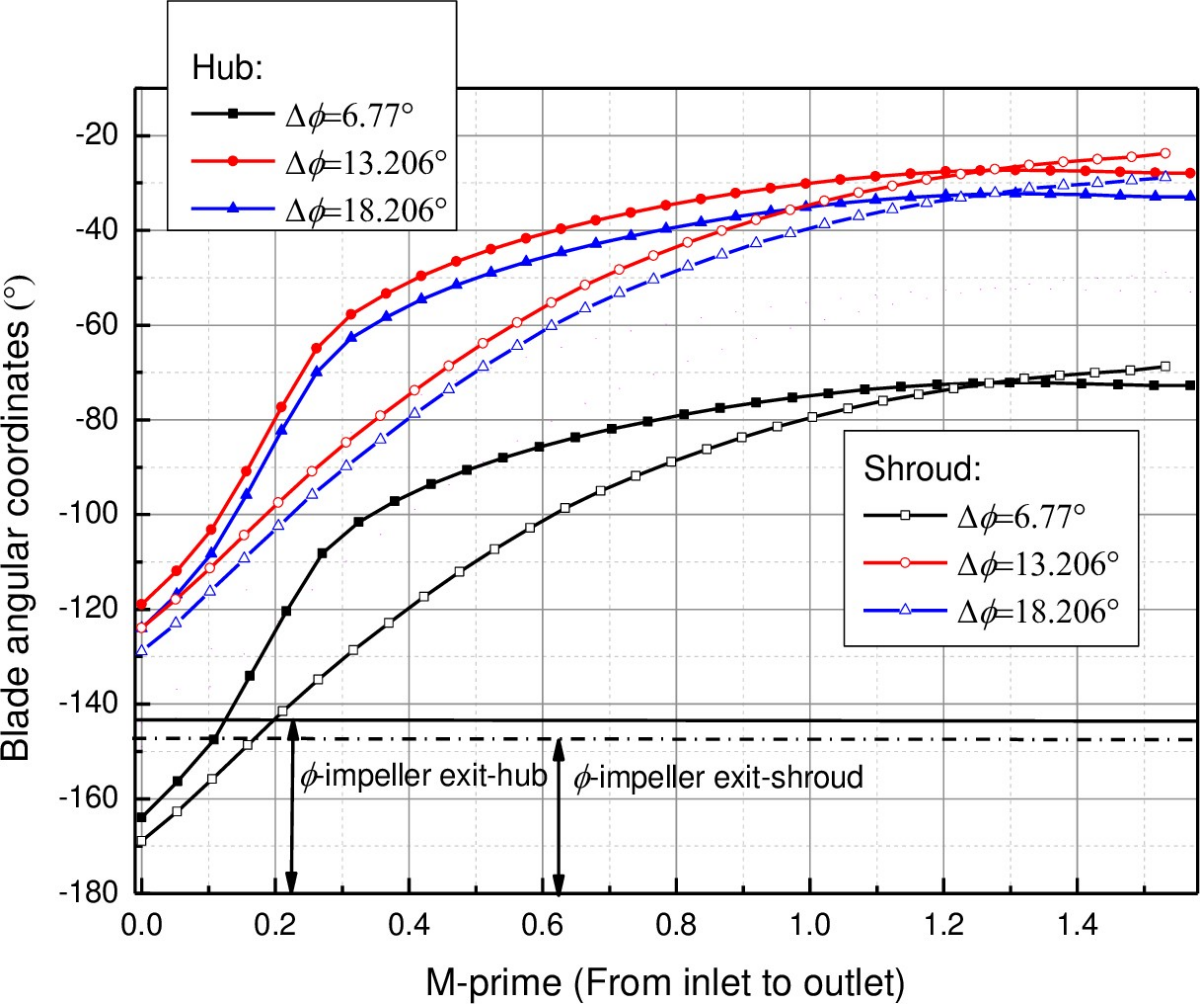
Blade angular coordinate distributions along with meridian streamline position.

The difference between the blade angular coordinates *ϕ* of the LE at the hub layer of the diffuser vane and the impeller exit (Δ*ϕ* = *ϕ*_*i*2_-*ϕ*_*d*1_) is adopted to describe the relative location of the diffuser vane to the impeller blade (see Fig 3). The blade angular coordinates of the diffuser is shown in Fig 4 and the blade angular coordinates of the impeller exit are -157.31° and -158.19° at the hub layer and the shroud layer, respectively. Meanwhile, it can be seen that the blade angular coordinates could be adjusted with the change of the vane position. In this case, taking the periodicity of the blade arrangement into consideration, pump performances for models with three different values of Δ*ϕ* (6.77°, 13.206° and 18.206°) are chosen for following discussions.

## 3. Numerical model and analysis strategy

### 3.1.Governing equation

Numerical analyses of the pump were carried out using the commercial software ANSYS CFX. On the basis of continuity equation and viscous fluid kinetic equation [10], the turbulence model used is the RNG *k-ε* model to solve the turbulence formulation [11]. The control equations were provided below, including the continuity equation, the momentum equation and the RNG *k-ε* turbulence equations (see Eqs (3)–(6)). All the above equations were discretized by finite volume approximations and solved on hexahedral grids.

$$\frac{\partial \rho }{\partial t}+\nabla \cdot (\rho \vec{u})=0$$ (3)

$$\frac{\partial \left(\rho \vec{u}\right)}{\partial t}+\nabla \cdot (\rho \vec{u}\vec{u})=-\nabla \cdot \left[\left(\mu +\mu_{t})(\nabla \vec{u}+\nabla {\vec{u}}^{T}\right)\right]-\nabla p+\rho \vec{g}$$ (4)

$$\frac{\partial }{\partial t}\left(\rho k\right)+\frac{\partial }{\partial x_{i}}\left(\rho ku_{i}\right)=\frac{\partial }{\partial x_{j}}\left[\left(\mu +\frac{\mu_{t}}{\sigma_{k}}\right)\frac{\partial k}{\partial x_{j}}\right]+G_{k}+G_{b}-\rho \varepsilon +S_{k}$$ (5)

$$\frac{\partial }{\partial t}\left(\rho \varepsilon \right)+\frac{\partial }{\partial x_{i}}\left(\rho \varepsilon u_{i}\right)=\frac{\partial }{\partial x_{j}}\left[\left(\mu +\frac{\mu_{t}}{\sigma_{\varepsilon }}\right)\frac{\partial \varepsilon }{\partial x_{j}}\right]+C_{1\varepsilon }\frac{\varepsilon }{k}\left(G_{k}+C_{3\varepsilon }G_{b}\right)-C_{2\varepsilon }\rho \frac{\varepsilon^{2}}{k}+S_{\varepsilon }$$ (6)

### 3.2 Mesh discretization

**Fig 5:**
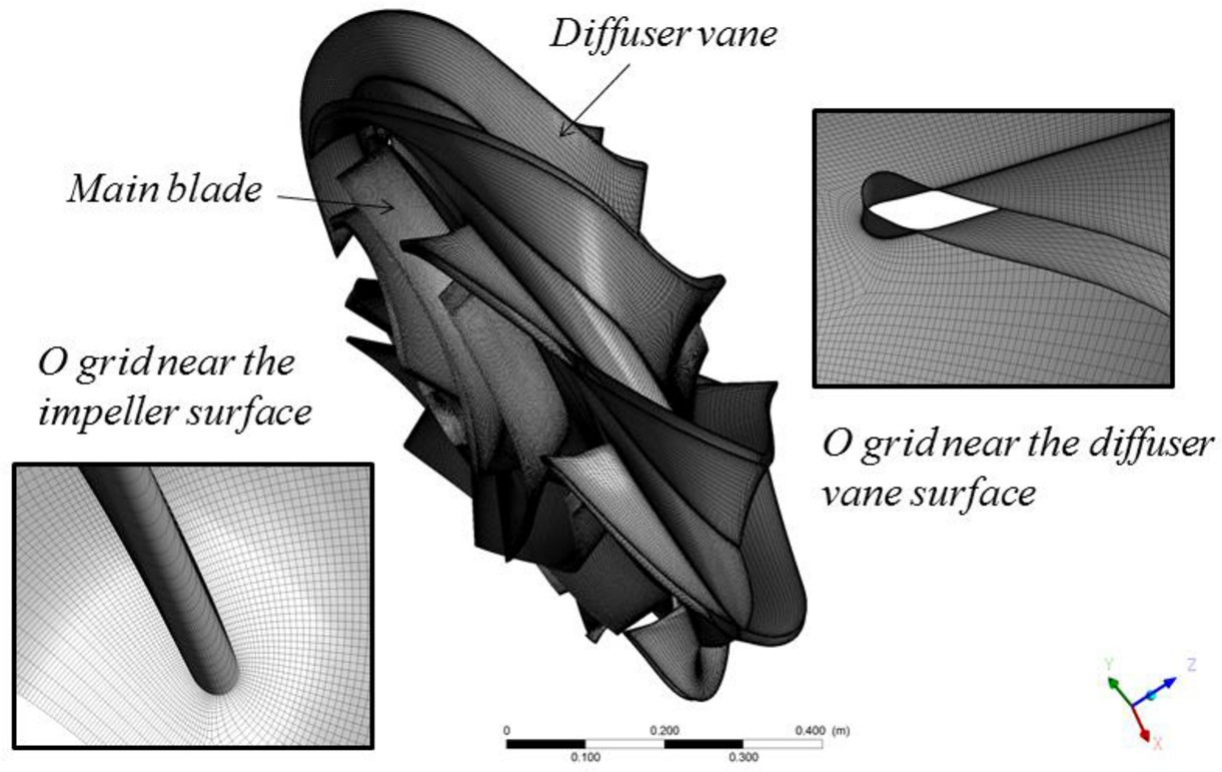
The mesh model of the impeller and the diffuser.

The hexahedral grids were generated using Turbo-Grid software and the grids of the impeller and the space diffuser are shown in Fig 5. Two mesh models (i.e. Mesh models A and B) with different element numbers are conducted to exclude the influence of grid on calculation accuracy (see Table 2). Taking the design point as an example, the water head and the efficiency of the pump are calculated and the results are shown in Table 3. It can be observed that all the errors of the pump heads and the efficiencies by the two mesh models are less than 5%. To gain more accurate results, mesh model B is selected for the following calculations, with the element number over 1.5*10^7^. Moreover, to ensure the quality of wall mesh, the values of the parameter *y*+ near the boundary wall are set as 90 for the impeller and 50 for the diffuser, which has been proven that the refined mesh model could match the turbulence model and the wall function adopted [12].

**Table 2:** Mesh information of different domains.

Mesh model	Inlet duct	Impeller	Space diffuser	Draft tube	All
A	1,414,880	2,307,998	2,647,679	1,042,668	7,413,225
B	2,022,661	3,979,255	4,834,662	4,527,354	15,363,932

**Table 3:** Pump head and pump efficiency calculated by different mesh models.

Mesh model	Pump head (m)			Pump efficiency (%)		
	Calculated	Tested	Error	Calculated	Tested	Error
A	60.02	57.54	4.29%	83.69%	79.72%	4.98%
B	59.21		2.90%	82.13%		3.02%

### 3.3 Solution strategies

The boundary conditions, solver governing equations, and post processing of the results were performed using ANSYS CFX and CFD-Post, respectively [3]. The inlet total pressure and the outlet mass flow rate were set as the inlet/outlet boundary conditions. Due to numerous industries relying not only on the operation of pumping systems with different required operating flow rates but also on the design flow rate [13], every case was carried out in several working conditions with flow rates ranging from 0.8*Q* to 1.13*Q* during the CFD analyses. No-slip boundaries were set in the computational domain and the scalable wall function was adopted to calculate the turbulent flow in the near wall [14]. The frozen rotor method was used for the interfaces between the rotary and stationary components [15–16], while those between two stationary components were set as the general grid. The advection scheme was set to high resolution for better precision and the convergence criterion for solution of the momentum and continuity equations was set as 10^−6^[17].

## 4. Results and discussion

### 4.1 Pump performance

**Fig 6:**
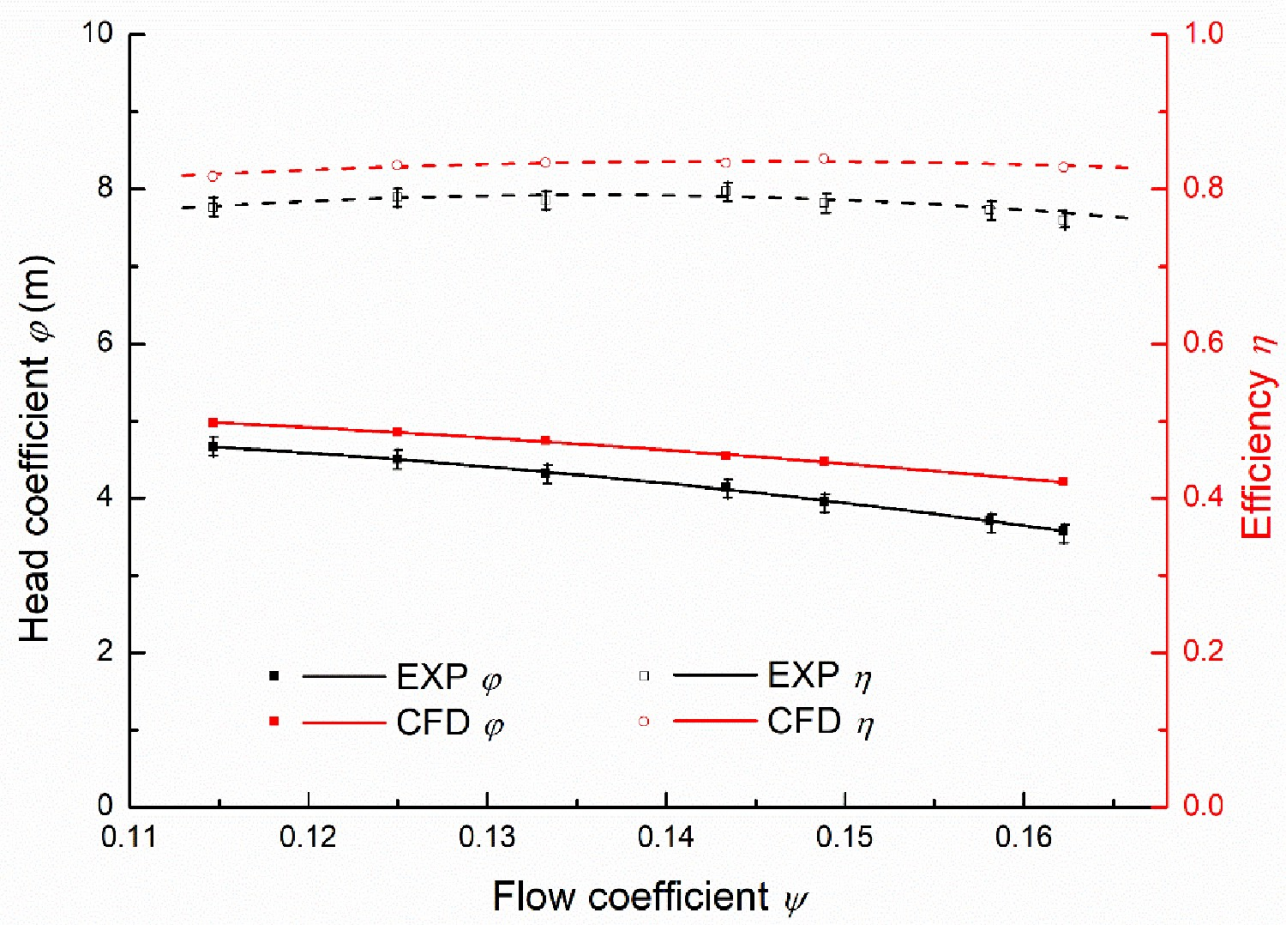
Experimental validation of the pump performance.

The pump model of the diffuser with the wrap angle of 110° is selected for the validation of the numerical results. Comparisons between the experimental and numerical results are shown in Fig 6. The dimensionless flow coefficient *ψ* and the head coefficient *φ* are defined as Eqs (7)–(8), respectively [18]. The flow coefficient and the head coefficient for the BEP were 0.14335 and 4.55, respectively. It can be seen that the predicted results of the pump present the similar trend to the experimental data, including the pump efficiency and the pressure head. It should be noted that the calculated flow efficiency and the pressure head are slightly higher than the experimental data. The over-prediction may be attributed to the neglecting of the leakage loss induced by the surface roughness, the clearances in pumps, and the mechanical loss caused by mechanical seals and bearings [19]. As a whole, the numerical method adopted in this work is reasonable for performance prediction of the pump.

$$\psi =\frac{Q}{nD_{2}^{3}}$$ (7)

$$\varphi =\frac{Hg}{n^{2}D_{2}^{2}}$$ (8)

### 4.2 Effect of the wrap angle of the diffuser

#### 4.2.1Performance curve

**Fig 7:**
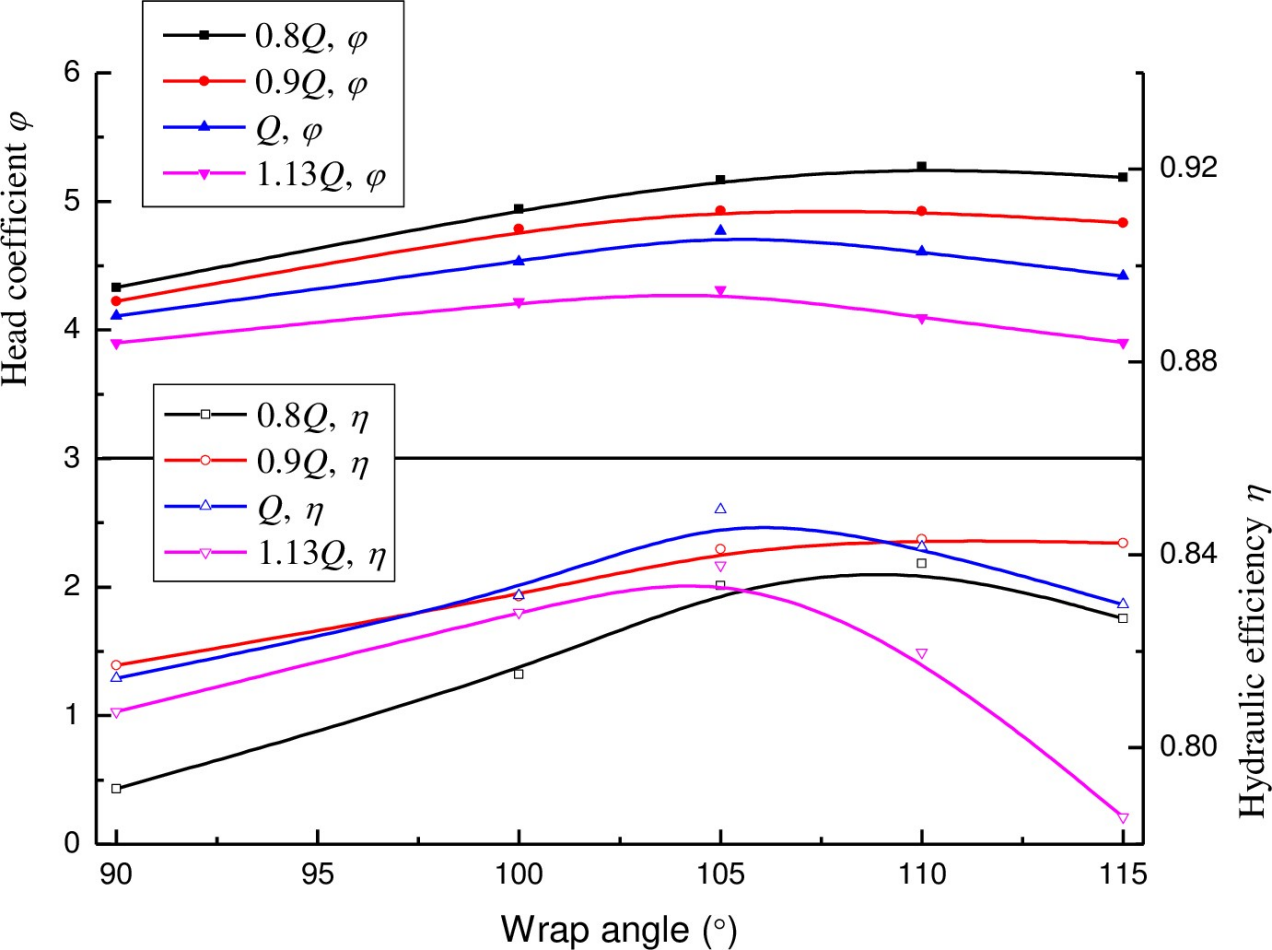
Pump performances for diffusers with various wrap angles.

The performance curves of the pump with different wrap angles of the diffuser are presented in Fig 7. It can be seen the wrap angle of the diffuser has a remarkable effect on the pump efficiency and pump head. Meanwhile, there is a best wrap angle of the diffuser, where the pump has the largest pump head and hydraulic efficiency. However, the best wrap angle is related to working conditions. For instance, the best wrap angle is 105° for the design point (i.e. Q) and a larger flow rate (e.g. 1.13Q), while it is 110° for a smaller flow rate (e.g. 0.8Q and 0.9Q). As a whole, for the design point, a suitable wrap angle of the diffuser vane may improve the pump hydraulic efficiency and the head by approximately more than 4% and 8%, respectively.

#### 4.2.2 Pressure recovery coefficient of the diffuser

**Fig 8:**
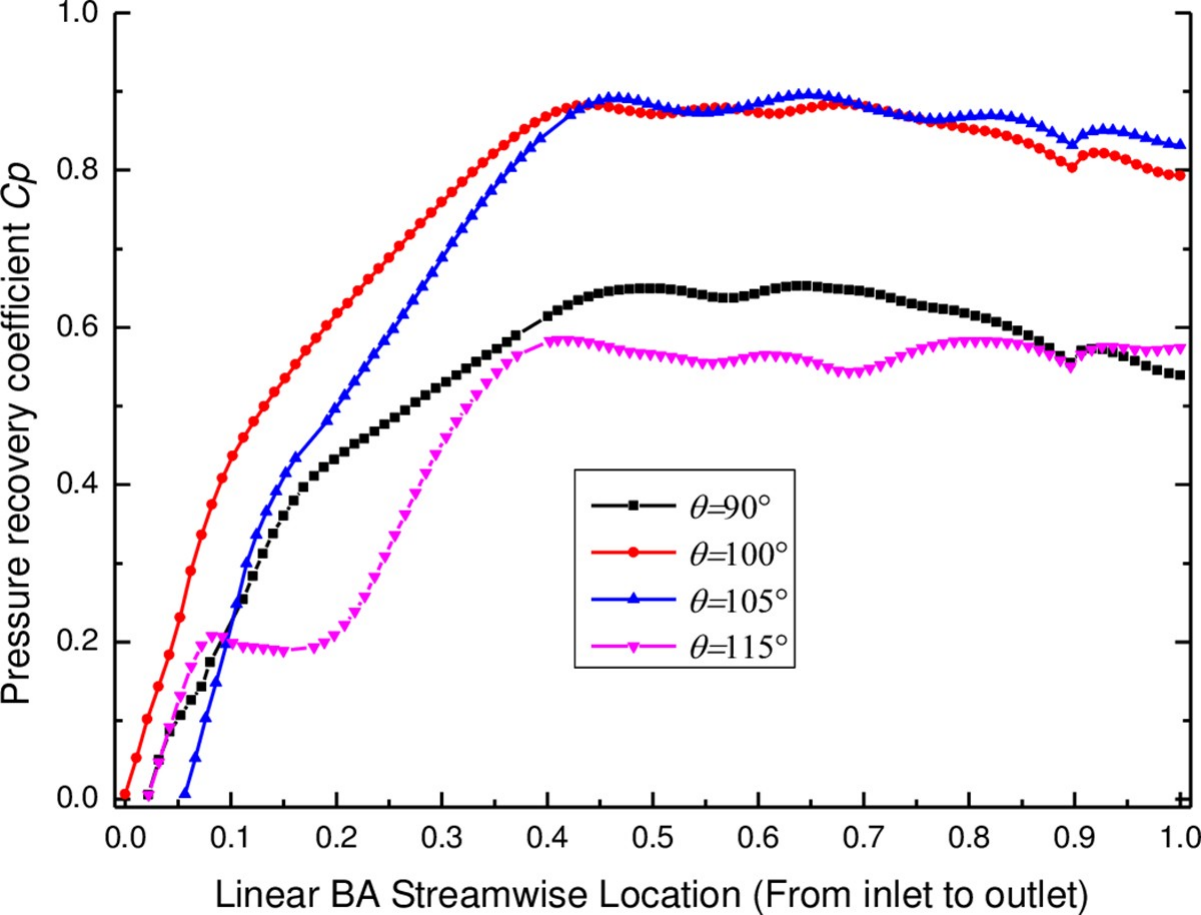
Pressure recovery coefficient of the diffusers with various wrap angles.

**Fig 9:**
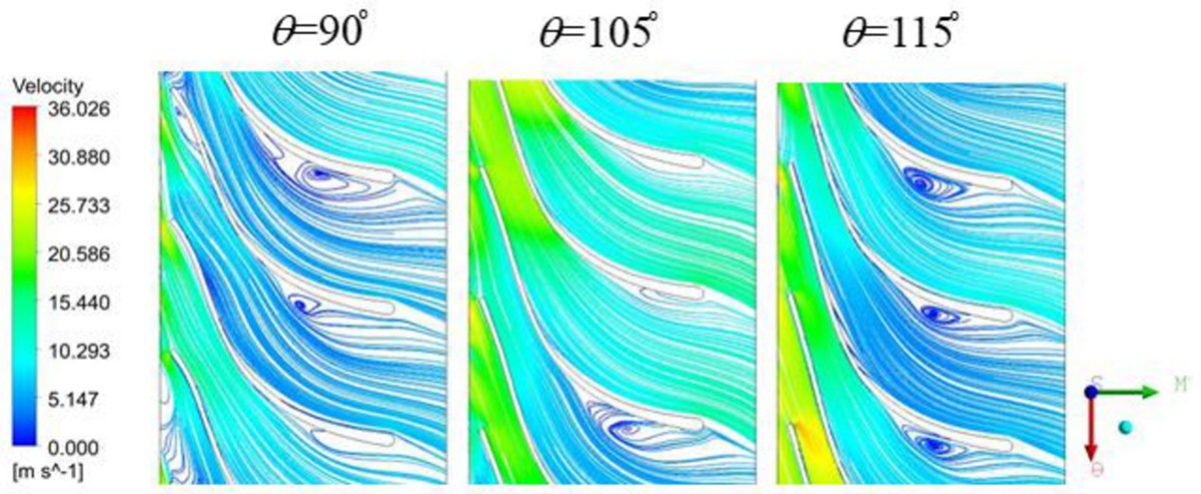
Shapes of the diffuser vanes with various wrap angles in Blade to Blade view.

Pressure recovery coefficients of the diffuser with various wrap angles are provided in Fig 8. The pressure recovery coefficient *C*_*p*_ is defined as the static pressure rise through the diffuser divided by the inlet dynamic pressure (see Eq (9)), which is one of the most frequently used parameter of the diffuser [20–21], indicating how much the dynamic energy at the inlet of the diffuser is converted into the static pressure by the diffuser vane. As seen in Fig 8, the pressure recovery coefficient increases significantly along the blade-aligned (BA) streamline direction from the position of 0 to 0.45, but then fluctuates slightly. Compared to the pump performance in Fig 7, the distribution of the pressure recovery coefficient of the diffuser at the trailing edge (TE) is closely related to pump performance. Meanwhile, a larger pressure recovery coefficient of the diffuser at the TE corresponds to a higher pump performance. For instance, the pressure recovery coefficient of the diffuser at the TE is the largest with the wrap angle of 105°, corresponding to the highest pump performance. At this time, the diffuser has a better pressurizing effect. In addition, the pressure recovery coefficient plotted for the wrap angle of 115^o^ shows wavered trend when compared to other trend lines (see Fig 8), which may result from the shape change of the diffuser vane. The vane shapes of the diffusers with various wrap angles are compared with each other in blade to blade view (see Fig 9) and it can be observed that the wrap angle changes the whole shape of the diffuser vane, including the LE and the TE shapes of the diffuse vane. When the wrap angle of the diffuser increases to 115^o^, the blade angles of the diffuser vanes nearly remain unchanged at a zone near the leading edge and this zone may cover the streamline locations that the wavered trend of the pressure recovery curve presents (see Fig 9).

$$C_{p}=\frac{p_{s}-p_{sin}}{0.5\rho v_{in}^{2}}$$ (9)

**Fig 10:**
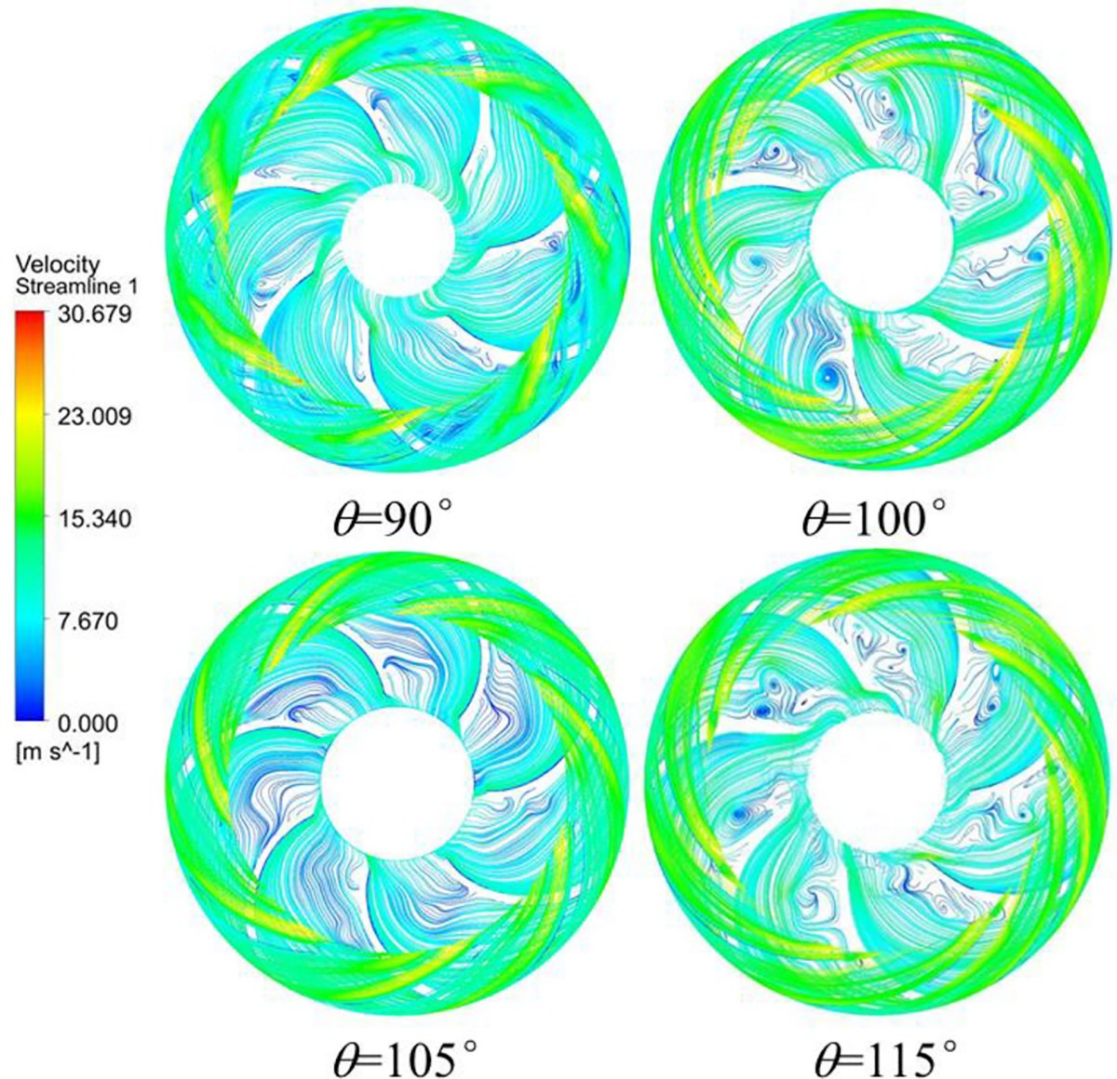
Velocity and streamlines of the diffusers with various wrap angles.

To show the influence of the wrap angle on the flow clearly, the internal flow characteristics of diffusers with various warp angles are also given in Fig 10. As the shapes of diffuser vanes change, the whole internal flow characteristic of the diffuser also presents some differences accordingly. More concretely, a large amount of vortex flow occurs at the TE position of the vane when the wrap angle of the diffuser vane is 115° and some vortexes also appear at the LE position of the vane when the wrap angle of the diffuser vane is 90°. Although a little vortex is found at the TE position of the vane when the wrap angle of the diffuser vane is 105°, the internal flow of the diffuser presents a better behavior at this wrap angle.

#### 4.2.3 Hydraulic loss of the pump

Water friction, collision, and vortex and flow separation in the pump model may lead to the hydraulic loss of the pump, which can also be adopted to explain the pump performance. The hydraulic losses of the pump can be calculated by Eqs (10)–(12). The subscripts 1 and 2 stand for the inlet and the outlet of the component and the subscript *i* represents the number of different parts. In general, the total hydraulic loss includes two parts: (1) the impact losses between two adjacent parts, such as the inlet pipe versus the impeller and the impeller versus the diffuser, the diffuser versus the outlet pipe; (2) the internal hydraulic loss of each component.

$$h_{s}=\frac{p_{i1}-p_{i2}}{\rho g}$$ (10)

$$h_{i}=\frac{p_{i1}-p_{(i-1)2}}{\rho g}$$ (11)

$$H=H_{t}-\sum h$$ (12)

**Fig 11:**
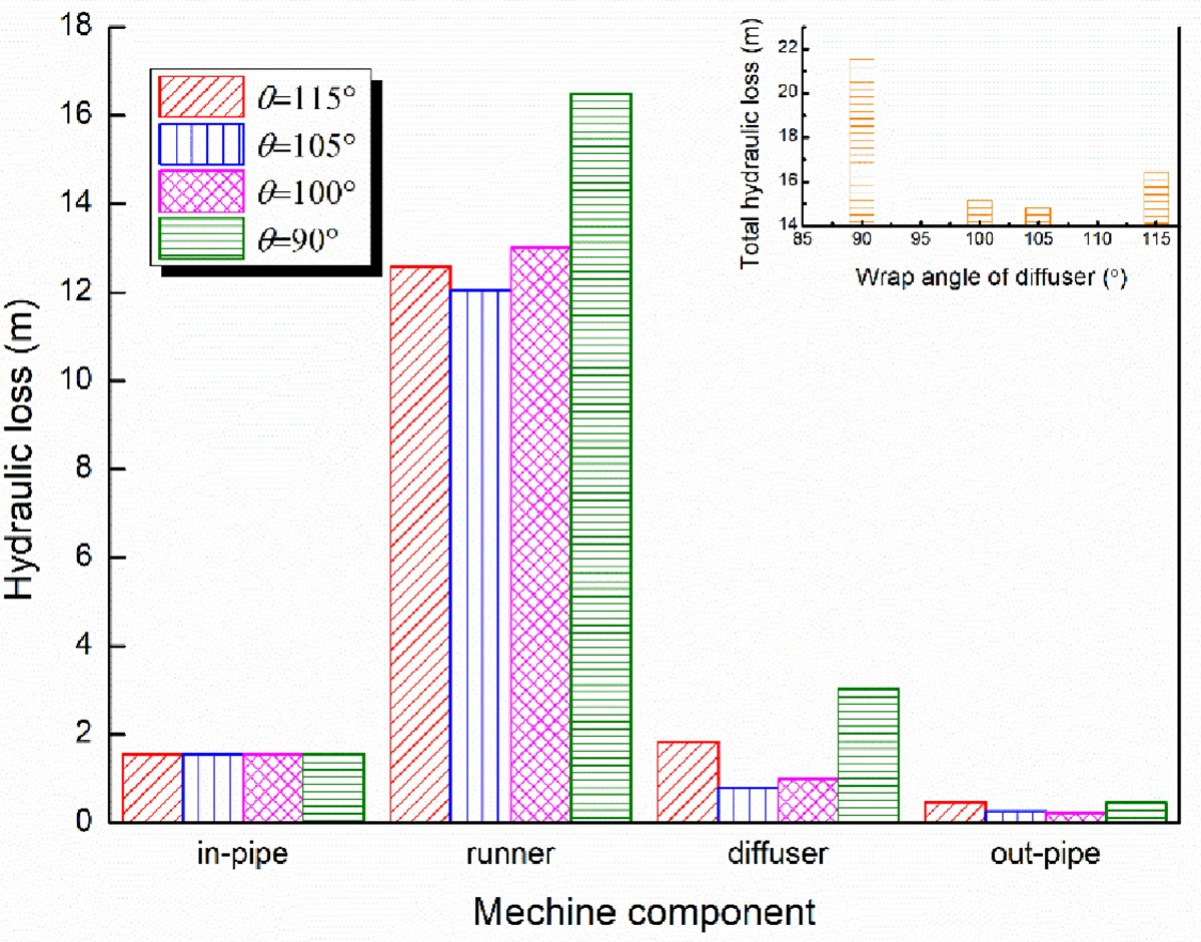
The hydraulic loss of the pump with various wrap angles of the diffuser.

The hydraulic losses of each component and the total hydraulic losses of the pump with various wrap angles of the diffuser are displayed in Fig 11. It can be observed that for the total hydraulic loss, the model with wrap angel of the diffuser *θ* = 105° indicates the smallest hydraulic loss for the conditions mentioned. Meanwhile, it should be noted that the hydraulic loss of the impeller is the largest among the four hydraulic components. This is because the impeller is the rotating part of the pump and the water friction in the impeller is larger due to the rotation effect. Meanwhile, the hydraulic loss of the impeller is also affected by the structural configuration of the diffuser. As mentioned in Section 2.2, various wrap angles of the diffuser could affect the distribution of the blade angles at the same time and the blade angle of the diffuser inlet determines the flow match from the impeller to the diffuser, then resulting in the loss of the adjacent component (i.e. impeller) directly connected with the diffuser.

#### 4.2.4 Local Euler head distribution

In recent years, 3D inverse design method proposed by Zangeneh [22] is adopted by many researchers and the blade loading is used to define the modification of the pump impeller and diffuser. The pressure loading (*p*^+^-*p*^-^: the pressure difference across the blade) is calculated through the following equation for incompressible flows. As shown in Eq (13), the blade pressure loading is directly proportional to distribution of ∂(*rV*_*θ*_)/∂*m*. And, the local Euler head is defined as the product of the local peripheral speed and the circumferential component of absolute velocity divided by the gravity acceleration (given in Eq (14)) [3], which has the same distribution with the expression of *rV*_*θ*_. Thus, the distribution of the local Euler head along the meridional direction could be adopted to describe the blade loading. During the calculation of the flow field of the pump, the expression and variables of Eq (14) can be defined through the CFX command language (CCL) and obtained by the CFD-Post. In addition, it should be noted that for centrifugal pumps, Euler head is adopted to describe the relationship between the theoretical head of the pump and the distribution of flow velocities at the inlet/outlet of the pump. The theoretical head of the pump increases with the rising flow velocities from the inlet to the outlet. In this paper, the definition of the local Euler head of each hydraulic component is similar to the total Euler head of the pump and the local Euler head shows the velocity changes in hydraulic components.

$$p^{+}-p^{-}=(2\pi /N)\rho W_{mbl}\frac{\partial (rV_{\theta })}{\partial m}$$ (13)

$$\mathrm{Euler}=\frac{V_{\theta }U}{g}=\frac{V_{\theta }r\omega }{g}$$ (14)

**Fig 12:**
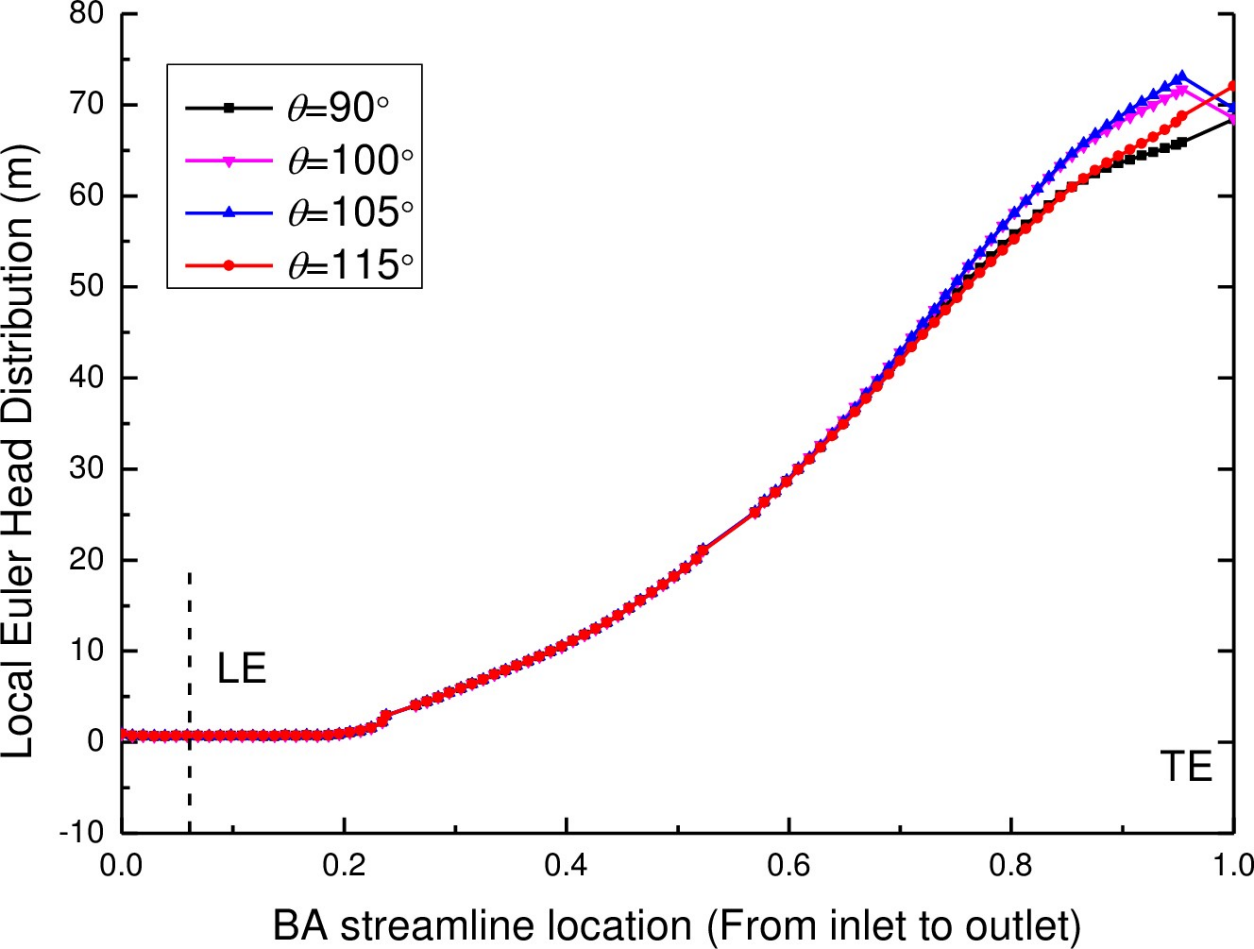
The local Euler head distribution of the impeller along the BA streamline location.

**Fig 13:**
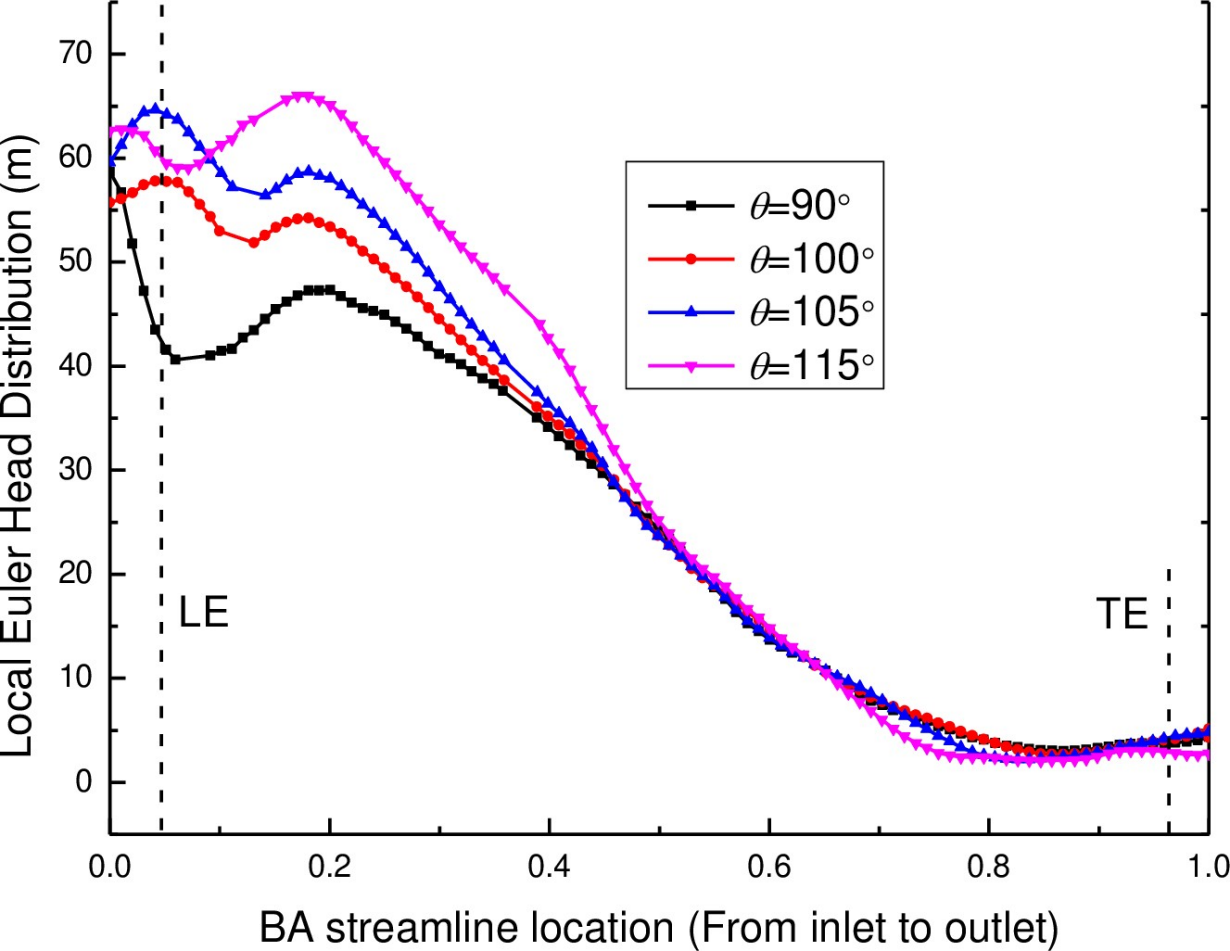
Local Euler head distribution of the diffuser along the BA streamline location.

The local Euler head of the pump components (including the impeller and the diffuser) with various wrap angles of the diffuser are shown in Figs 12 and 13. It can be seen that the local Euler head of the impeller from the inlet to the BA streamline location of 0.2 keeps the same value of approximately zero and then increases along the stream wise direction, which means a rising velocity component and a rising theoretical head of the impeller from the inlet to the outlet of the impeller. For various wrap angles of the diffuser, the distributions of the local Euler head remain the same for the zone from the impeller inlet to the BA streamline location of 0.75 but show different distributions for the zone from the BA streamline location of 0.75 to the impeller outlet. It can be found that the local Euler head has a decrease near the TE of the impeller when the wrap angles are 105° and 100°, at which the pump has a high efficiency. Moreover, for the local Euler heads with the wrap angles of 105° and 100°, the hydraulic efficiency is higher for a larger peak value of the local Euler head.

As displayed in Fig 13, there are larges differences in the local Euler head of the diffuser near the leading edge of the diffuser from the inlet to the BA streamline location of 0.2 but then decreases along the stream wise direction. This may result from the function of the diffuser: eliminating the velocity of the rotation component and transforming the velocity energy to the pressure energy. For various wrap angles of the diffuser, it has a great effect on the local Euler head distribution of the diffuser itself, especially at the position from the inlet to the half position of the blade. By comparison, the local Euler heads have peak values and the change rate of the local Euler head is zero at the leading edge of the diffuser when wrap angles of the diffuser are 105° and 100°, at which the pump has a high hydraulic efficiency. Zero change rate of the local Euler head means no blade loading at the diffuser leading edge. This conclusion is consistent with the conclusion found by Goto and Zangeneh [9] that no blade loading at the diffuser leading edge ensures the best match between impellers and diffusers.

### 4.3 Relative location of the diffuser vane

#### 4.3.1 Performance curve of the pump

**Fig 14:**
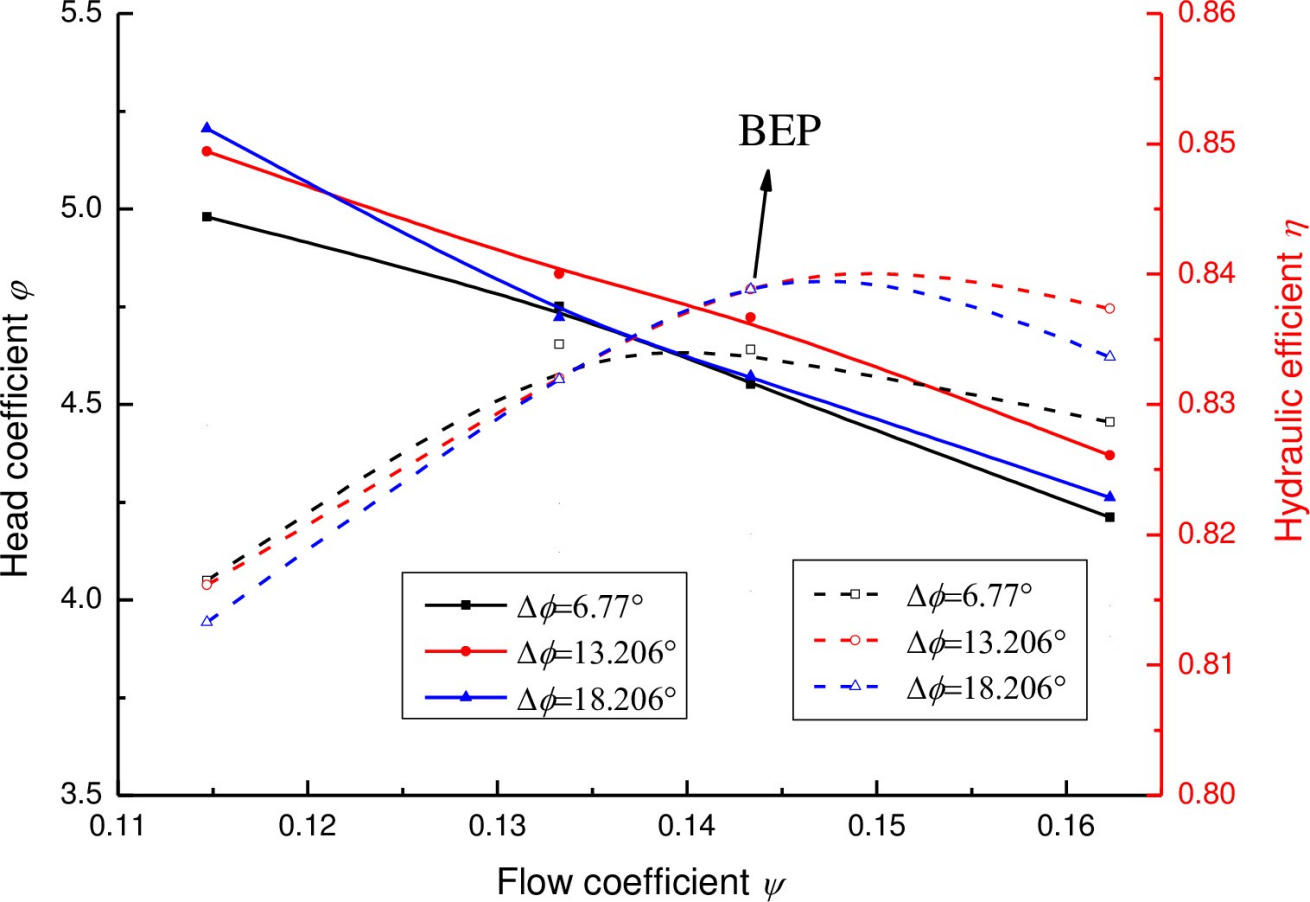
Performance curves of the pump under various positions of diffuser vane.

The performance curves of the pump with various relative positions of the diffuser vane to the impeller blade are displayed in Fig 14. It can be observed that the relative position of the diffuser vane to the impeller blade has a prominent effect on the pump efficiency and pump head. There is a best position of the diffuser for pump performance but the exact position is different for various working conditions. Regarding the BEP, the efficiencies of the pump are the same when the relative angle of the diffuser vane to the impeller blade is Δ*ϕ* = 13.206° and Δ*ϕ* = 18.206°. But the pump head for the angle Δ*ϕ* = 13.206° is 4% higher than that for Δ*ϕ* = 18.206°. Regarding a higher flow rate (e.g. 1.13*Q*), the hydraulic efficiency and pump head for Δ*ϕ* = 13.206° are both higher than that for Δ*ϕ* = 6.77° and Δ*ϕ* = 18.206°. It can be concluded that for the BEP and a higher flow rate (e.g. 1.13*Q*), Δ*ϕ* = 13.206° is the best position of the diffuser for pump performance but it is Δ*ϕ* = 6.77° for a smaller flow rate (e.g. 0.8*Q* and 0.9*Q*).

#### 4.3.2 Pressure recovery coefficient of the diffuser

**Fig 15:**
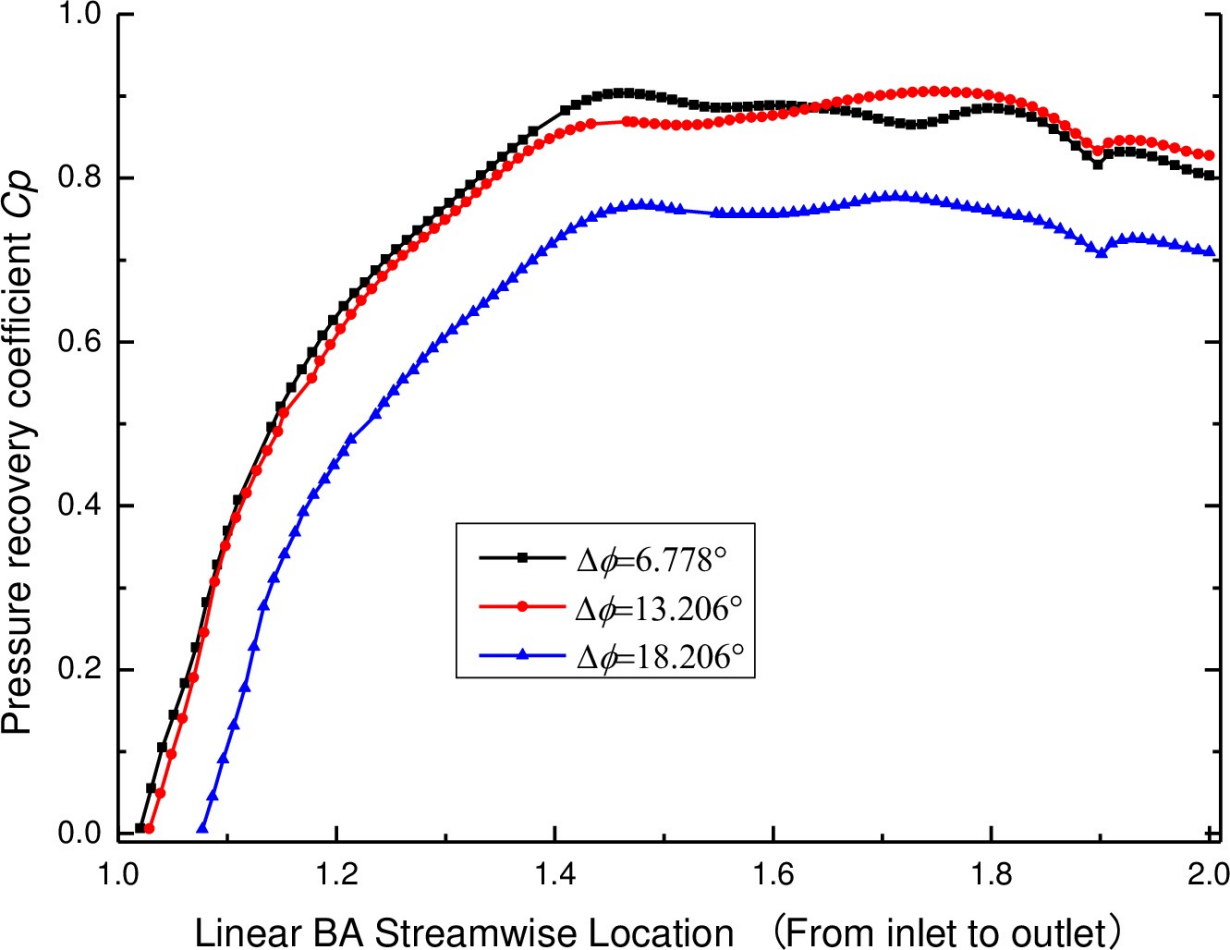
Pressure recovery coefficients of the diffusers with various vane positions.

The design point with the best positive angle of 13.206° is taken to illustrate why the diffuser has the best relative position to the impeller for a given condition. The pressure recovery coefficients of the diffusers are displayed in Fig 15. It can be seen that the pressure recovery coefficient rises from the inlet to the half blade of the diffuser but then fluctuates near the largest value. By comparing the pressure recovery coefficients at different vane positions, it can be observed that the pressure recovery coefficient has a high value at the diffuser outlet, indicating that this diffuser has a better pressurizing effect.

#### 4.3.3 Hydraulic loss of the pump

**Fig 16:**
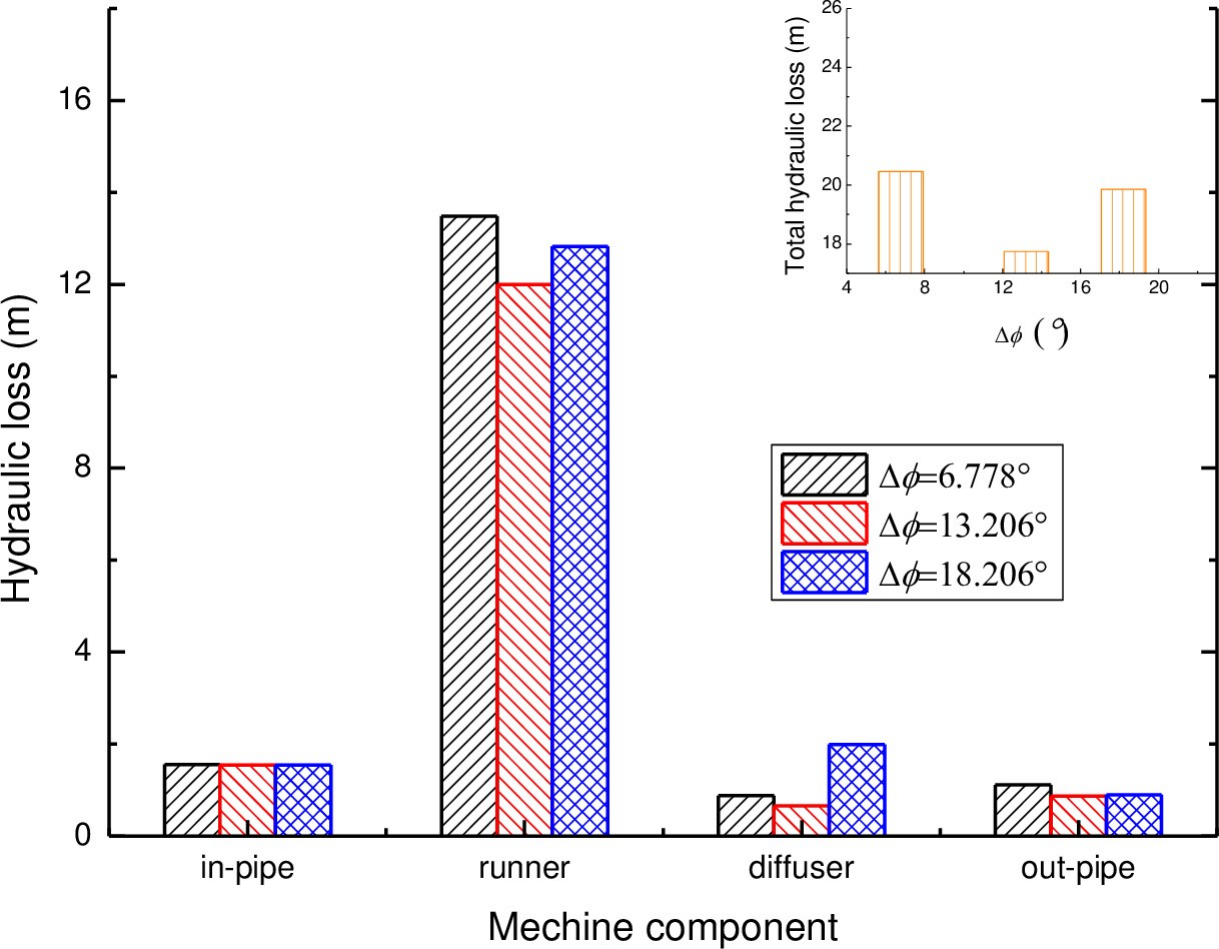
The hydraulic loss of the pump with various vane positions of the diffuser.

The hydraulic losses of each component and the total hydraulic losses of the pump with various vane positions of the diffuser are shown in Fig 16. It can be seen that for the hydraulic loss, the model with the relative position angel of Δ*ϕ* = 13.206° displays a smaller hydraulic loss in three main components of the pump, such as the impeller, diffuser and the outlet pipe. As a whole, the model with the relative position angel of Δ*ϕ* = 13.206° also indicates the smallest hydraulic loss for the conditions mentioned, contributing to a better pump performance. This indicates that a good position matching of the blades of the impeller and the vanes of the diffuser may make the flow more smooth and reduce the secondary flow and the eddy, and then the hydraulic loss of the pump is decreased.

#### 4.3.4 Local Euler head distribution

**Fig 17:**
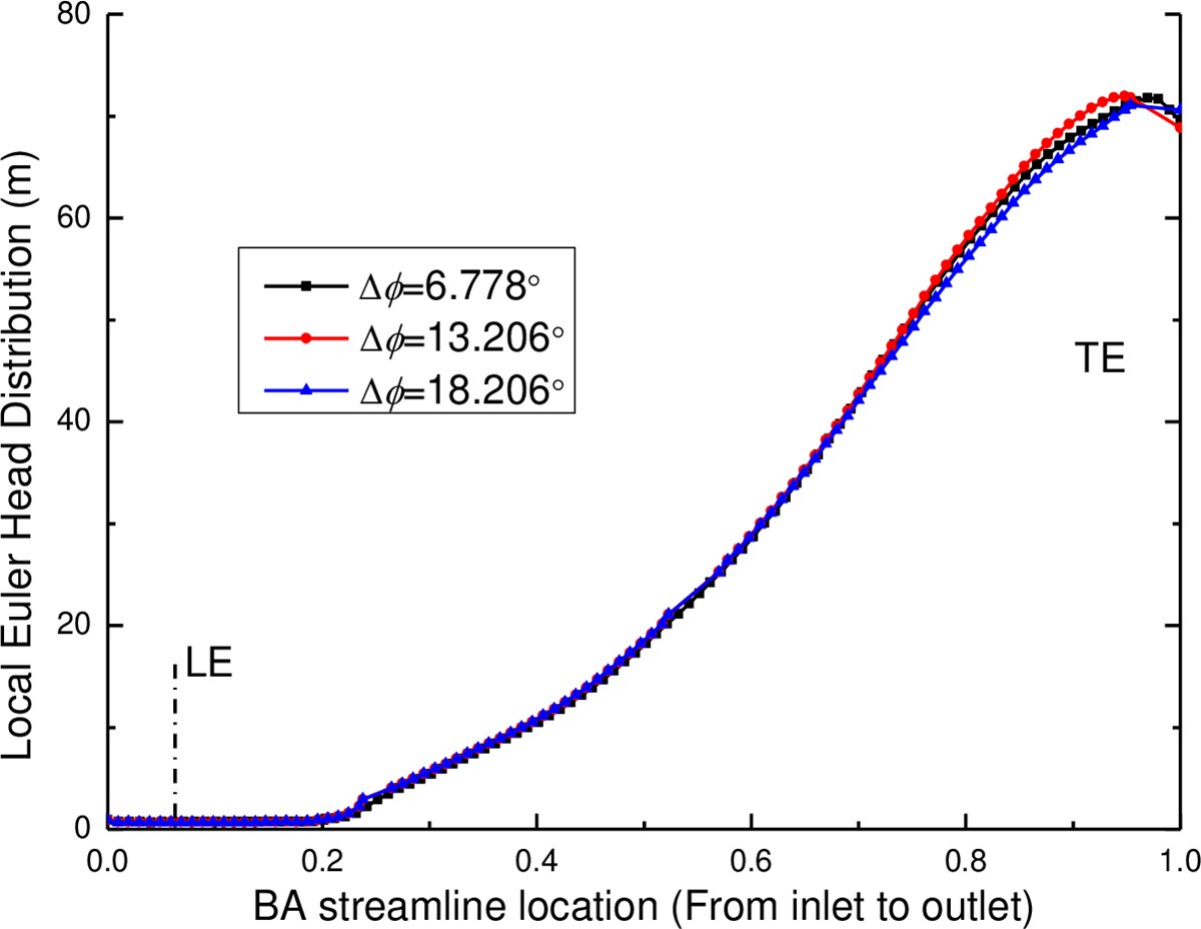
Local Euler head distributions of the impeller along the BA streamline location.

**Fig 18:**
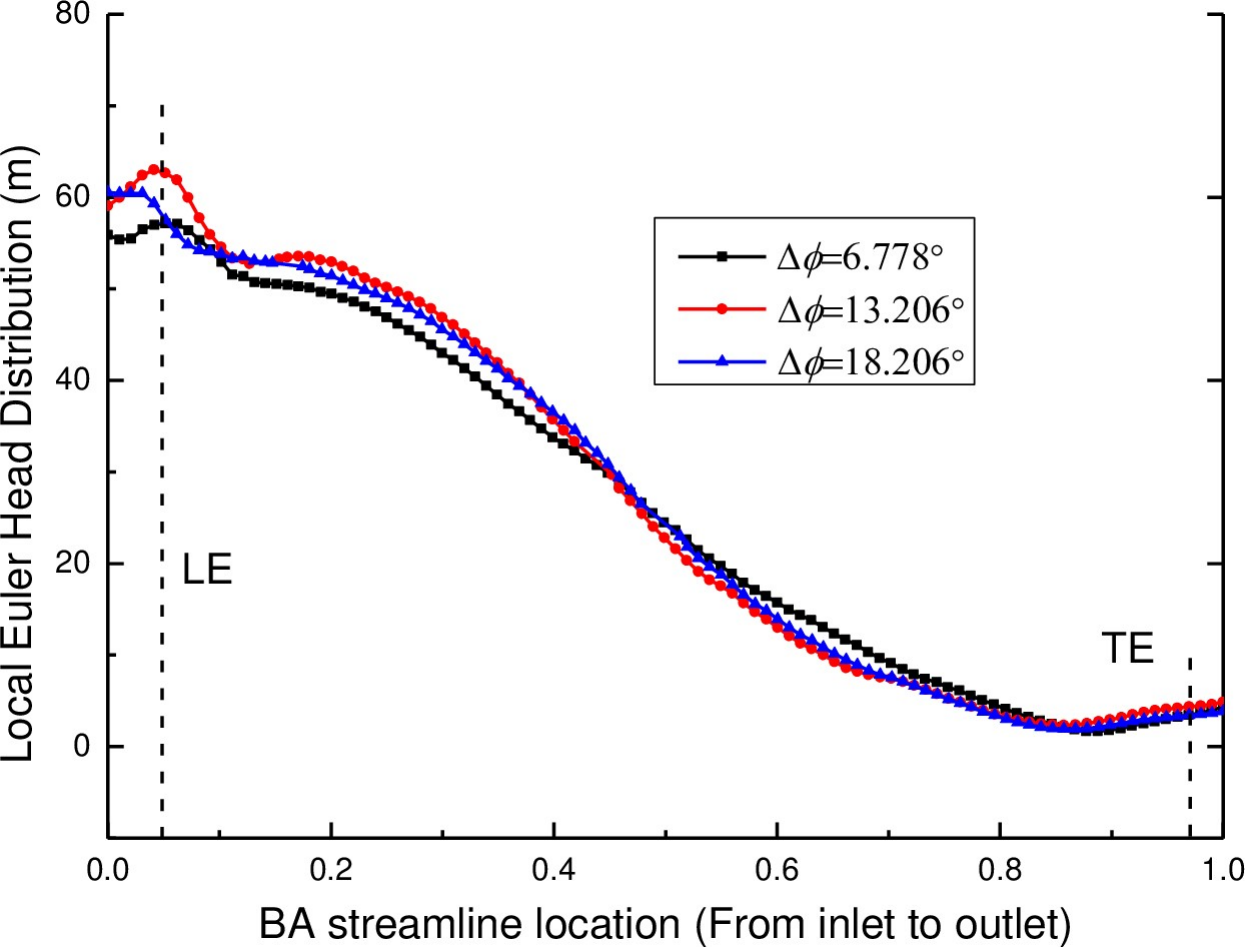
Local Euler head distributions of the diffuser along the BA streamline location.

The local Euler head distributions of the pump components with various relative positions of the diffuser vane to the impeller blades are shown in Figs 17 and 18, including the impeller and the diffuser. As displayed in Fig 17, the local Euler head distribution of the impeller with various relative positions of the diffuser vane to the impeller blades is similar to that in Fig 12. The distributions of the local Euler head keep the same for the zone from the impeller inlet to the BA streamline location of 0.75 but show different distributions for the zone from the BA streamline location of 0.75 to the impeller outlet. However, the local Euler head of the impeller has a decrease near the trailing edge of the impeller when the relative position angle is 13.206°. Meanwhile, the peak value of the local Euler head of the impeller is the largest when the relative position angle is 13.206°, at which the pump has a high hydraulic efficiency. As a whole, for a higher hydraulic pump performance, the local Euler head of the impeller increases along the BA streamline direction from the inlet to the outlet zone but has a decrease near the trailing edge of the impeller. At this position, the local Euler head of the impeller has a peak value and the pump performance is better for a larger peak value.

The local Euler head distributions of the diffuser along the BA streamline location are shown in Fig 18, similar to that in Fig 13. It can be observed that the local Euler head of the diffuser decreases along the stream wise direction except the position near the leading edge. And, for various relative positions of the diffuser vane, it has a great effect on the local Euler head especially at the position near the leading edge. When the relative angle of the diffuser vane is 13.206°, the local Euler head has a peak value at the leading edge and its change rate is zero at this position, indicating that the pump has a high hydraulic efficiency. As a whole, for a higher hydraulic pump performance, the local Euler head of the diffuser has a peak value at the leading edge with a change rate of zero along the BA streamline location. The zero change rate means that no blade loading at the diffuser leading edge ensures the best match between impellers and diffusers.

## 5. Conclusion

In this paper, the effects of some critical geometric parameters of the diffuser on pump performances were investigated for optimization by CFD codes, including the wrap angle and the position of the diffuser vane. Meanwhile, the pressure recovery coefficient and the local Euler head of the diffuser were introduced to evaluate the performance of the diffuser. The main conclusions drawn are given as follows:

1. An optimized configuration of the diffuser with high hydraulic efficiency and high pump head was obtained. For the design point, a suitable wrap angle (i.e. 105°) of the diffuser vane may improve the pump hydraulic efficiency and the head by approximately 4% and 8%, respectively. A suitable relative position of the diffuser (i.e. 13.206°) vane to the impeller may enhance the pump head by more than 4%.
2. The pressure recovery coefficient was proved to be adequate to evaluate the flow field of the diffuser. A larger pressure recovery coefficient at the trailing edge of the diffuser can be gained for a better diffuser performance.
3. The distribution of the local Euler head is closely related to the blade loadings and the pump performances. For a high pump performance, the local Euler head of the impeller increases along the BA streamline direction from the inlet to the outlet zone but has a decrease near the trailing edge of the impeller. For the local Euler head of the diffuser, it has a peak value at the leading edge with the change rate of zero along the BA streamline location, indicating that no blade loading at the leading edge of the diffuser guarantees a better match between the impeller and the diffuser.

### Data accessibility

Our data are deposited at Dryad: https://doi.org/10.5061/dryad.g79cnp5kv.

## Nomenclature

*A*_2_: Area of the diffuser exit, m^2^
C_p_: Pressure recovery coefficient
*C*_1ε_: Empirical coefficient
*C*_2ε_: Empirical coefficient
*D*_1_: Inlet Diameter of impeller, mm
*D*_2_: Outlet Diameter of impeller, mm
*D*_3_: Inlet Diameter of the diffuser, mm
g: Gravitational acceleration, m^2^/s
G_b_: Generation of turbulence kinetic energy due to buoyancy, J
G_k_: Generation of turbulence kinetic energy due to the mean velocity gradients, J
h_s_: Total hydraulic losses, m
h_s_: Internal hydraulic loss of each component, m
h_r_: Impact loss of each component, m
H: Pump head, m
H_t_: Theoretical pump head, m
k: Turbulent energy, J
m: Meridional distance, m
n: Rotation speed, r/min
N: Blade number
p: Pressure, Pa
p+: Pressure on the pressure surface of the impeller blade, Pa
p-: Pressure on the suction surface of the impeller blade, Pa
p_s_: Static pressure, Pa
p_sin_: Inlet static pressure, Pa
r: Radial coordinate of blade
t: Time, s
Q: Flow rate, m3/h
S_k_: Source terms of turbulent energy, J
S_ε_: Source terms of turbulent dissipation rate
u: Speed, m/s
*u*_2_: Peripheral speed at the diffuser exit, m/s
U: Circumferential component of absolute velocity, m/s
v_in_: Inlet velocity, m/s
V_θ_: Circumferential averaged swirl velocity, m/s
W_mbl_: Meridional component of a relative velocity on the blade
β: Blade flow angle
ε: Turbulent dissipation rate
θ: Wrap angle of the diffuser vane
σ: Turbulent Prandtl numbers
*σ*_ε_: Empirical coefficient
ρ: Density, kg/m^3^
ϕi1: Blade angular coordinates of the LE at the hub layer of the impeller exit
ϕd2: Blade angular coordinates of the LE at the hub layer of the diffuser vane
ψ: Flow rate coefficient
φ: Head coefficient
μ: Viscosity, Pa S
μ_t_: Turbulent viscosity, Pa S
